# Teaching and performing audits on caesarean delivery reduce the caesarean delivery rate

**DOI:** 10.1371/journal.pone.0202475

**Published:** 2018-08-27

**Authors:** Emmanuelle Lesieur, Julie Blanc, Anderson Loundou, Arnaud Claquin, Michele Marcot, Helene Heckenroth, Florence Bretelle

**Affiliations:** 1 Department of Gynaecology and Obstetrics, Gynépole, Assistance Publique-Hôpitaux de Marseille, AMU, Aix- Marseille Université, Marseille, France; 2 EA 3279 –Public Health, chronic diseases and quality of life–Research Unit, Aix-Marseille University, Marseille, France; 3 Mediterannée Perinatal Networks « Reseau Mediterannée », PACA Corse Monaco, France; 4 UM63, CNRS 7278, IRD 198, INSERM 1095, Clinical infectious disease, Research Unit, Aix-Marseille University, Marseille, France; Massachusetts General Hospital, UNITED STATES

## Abstract

**Aim:**

To assess the factors associated with lower rate of caesarean deliveries in the South of France, based on the characteristics and organisation of the region’s 40 maternity facilities and the characteristics of the practitioners in these facilities.

**Method:**

A retrospective study from 1 January 2012 to 31 December 2015. Data were collected by the Mediterranean network and a declarative survey was completed by each maternity facility in the region to study factor which could be associated with lower caesarean rate by univariate and multivariate analysis.

**Results:**

250 564 women gave birth during this period, of which 55 097 by caesarean section. The mean caesarean delivery rate over the four years was 22.0%. The rate was significantly higher in private maternity facilities [23.9% (21.9%– 25.8%), p<0.05] and type III (maximum care level) maternity facilities [24.2% (21.3%– 27.1%), p<0.05]. After a stepwise regression, the factors associated with a decrease in the caesarean delivery rate were audits concerning caesarean delivery (19.83%, β = - 2.48, p = 0.03 over the four years) and the provision of training to trainee doctors at the maternity facility (20.28%, β = - 1.08, p = 0.04 over the four years).

**Conclusion:**

Performing audits in relation to caesarean deliveries could affect the caesarean. Teaching trainee doctors could be an indicator of quality of caesarean practices. They should be encouraged in maternity facilities to reduce the rate of caesareans.

## Introduction

Throughout the world in recent years, there has been a significant increase in the overall rate of caesarean deliveries [1]. In France, the caesarean rate rose from 10.9% in 1981 to 20.8% in 2013, then stabilized at 20.2% in 2016 [2,3]. Caesarean delivery rate above the WHO’s upper recommended rate of 15% [4] has not resulted with an improvement in neonatal outcomes but with an increase in maternal morbidity [5–7]. Indeed, caesarean sections lead to an appreciable increase in maternal complications in the short term (thromboembolism, infection, trauma, bleeding) and long term (risk of ectopic pregnancy, infertility, abnormal placental insertion, risk of uterine rupture) [8,9] and an increase in health costs [10].

The literature is now unanimous: the groups contributing the most to the overall caesarean section rate are Robson classification groups 1 and 2 (full-term first-time mothers with a single fetus presenting head first with induced or spontaneous labour) and group 5 (multiparous women with uterine scarring) [11]. A reflexion is so needed to optimize the management of these daily situations and to identify the factors associated with lower rates of caesarean section. Only one study has so far analysed and evaluated the factors and conditions that can reduce the caesarean delivery rate [12] and none have evaluated the impact of training to trainee doctors on the caesarean delivery rate.

The objective of our study was to identify the factors associated with lower rate of caesarean section, based on the maternity facilities’ organisation and the characteristics of the practitioners in these facilities.

## Materials and methods

### Data collection and study population

The first data was collected retrospectively from the PMSI (French National Computerized Medical Information System) by the Mediterranean network each year from 1 January 2012 to 31 December 2015. Our population included all births in the Provence-Alpes-Côte d'Azur region, Corsica and Monaco between 1 January 2012 and 31 December 2015, i.e. all living or stillborn children over 22 gestation weeks (GW) or weighing more than 500 g, born in private and public maternity facilities in the region.

The second data collection was from a declarative survey. The data was collected to qualify the practitioner or maternity facilities from a declarative process and was obtained by the maternity managers (midwife manager or obstetrician—head of maternity). This questionnaire completed in each of the 40 maternity facilities in the Provence-Alpes-Côte d'Azur region between 5 March and 15 April 2017. This questionnaire provided information on the characteristics of the practitioners and the internal organisation at the maternity facilities. Ethics approval was not required for the questionnaire portion of the study according to French law because information collected relates to the characteristics of maternity facilities and not personal data relating to patients.

### Studied factors

Firstly, the variables analysed focused on maternity unit characteristics: status (public health facilities or private health facilities, for-profit or not-for-profit facilities), level of care (Type I: without neonatal department, type IIa and IIb: with neonatal department and type III: with neonatal intensive care unit) and location. The mean overall caesarean rate in the Provence-Alpes-Côte d'Azur region was then calculated for each year from 2012 to 2015 based on these variables.

We then analysed the characteristics of the practitioners and the organisation of the maternity facilities including: the distance between the initial maternity facility and a university hospital, the size of the towns where the maternity facilities are located (small town: fewer than 20 000 inhabitants, medium town: 20 000 to 200 000 inhabitants, large town: more than 200 000 inhabitants), whether a protocol is in place for managing situations potentially leading to caesarean sections (fetal heart rhythm abnormalities, gestational diabetes and fetal macrosomia, occiput posterior fetal position, multiple pregnancies, breach presentation, uterine scarring, induction methods, elective caesarean section), and whether there are daily obstetrical staff meetings, assessments of professional practices (to analyse the clinical activity in a maternity, in relation to the professional recommendations, to improve the professional activity and the quality of the care: morbidity-mortality reviews, patient feedback, clinical pathway, relevance reviews, audit related to caesarean rates), second-line monitoring (scalp pH and/or lactate), any assistance provided during caesarean deliveries (scrub nurses, midwives, foundation doctors, or none), and whether maternity facilities offering training to trainee doctors. Data on participation in delivery room activities (more precisely to supervise in the delivery room and to intervene for deliveries requiring the presence of an obstetrician), participation in the on-call work, age and gender of each practitioner were also collected.

## Statistical analysis

The statistical analyses were conducted using the IBM SPSS Statistics software, version 20.0 (SPSS Inc., IL, USA). The continuous variables are presented as means +/- standard deviation or as a median with the interquartile range. The categorical variables are presented as numbers and percentages. These are binary variables where the references are 0 when the categorical variable is coded as 0/1 in the statistical table. The link between two qualitative variables was evaluated using Pearson’s chi-squared test or Fisher's exact test if the theoretical numbers were less than 5. The link between a quantitative variable and a two-category categorical variable was evaluated using Student’s t-test for independent samples. The link between two quantitative variables was evaluated using the Pearson correlation coefficient.

A multivariate analysis was performed over four years, using multiple linear regression and stepwise regression with the variables having an impact in the univariate analysis (size of the towns, protocol, assessment of professional practices, maternity offering training to trainee doctor, scalp lactate, age of each practitioner, gender of each practitioner, participation of each practitioner to delivery room, status and type). The multivariate model used models the caesarean rate by maternity facility, a quantitative variable, and not by patient. In this case, the results are presented with the β coefficient. For all tests, the statistical significance was set at p<0.05.

## Results

### Analysis of the overall caesarean delivery rate at each maternity facility

Over the four years, 250 564 women gave birth in the Provence-Alpes-Côte d'Azur region. The 55 097 caesarean sections were analysed, i.e. an overall mean caesarean rate of 22,0% over the four years throughout the Mediterranean network. The data from one maternity facility (southern Corsica) could not be used as it was incomplete. The analysis therefore focused on the 40 other maternity facilities in the Provence-Alpes-Côte d'Azur region. Thirteen maternity (32,5%) facilities were private and 27 (67,5%) were public. Twenty-two maternity facilities (55%) were type 1 (lowest level of care), 16 (40%) were type 2 (intermediate level of care) and two (5%) were type 3 (highest level of care) (AP-HM and Nice).

From 2012 to 2015, the overall caesarean delivery rate in the Provence-Alpes-Côte d'Azur region was, in chronological order, 22.1%, 22.7%, 21.6% and 21.6%, with no statistically significant difference between the four years (p = 0.84).

In 2012, 2013, 2014, and 2015, the lowest caesarean delivery rates in the Provence-Alpes-Côte d'Azur region were 11.9%, 10.8%, 13.8% and 10.7%, respectively, in a public level 1 maternity facility. The highest rates were 30.1%, 31.5%, 31.7% and 30.3%, respectively, in a private level 1 maternity facility. There was no significant difference in caesarean delivery rates between departments (p = 0.74).

**Fig 1:**
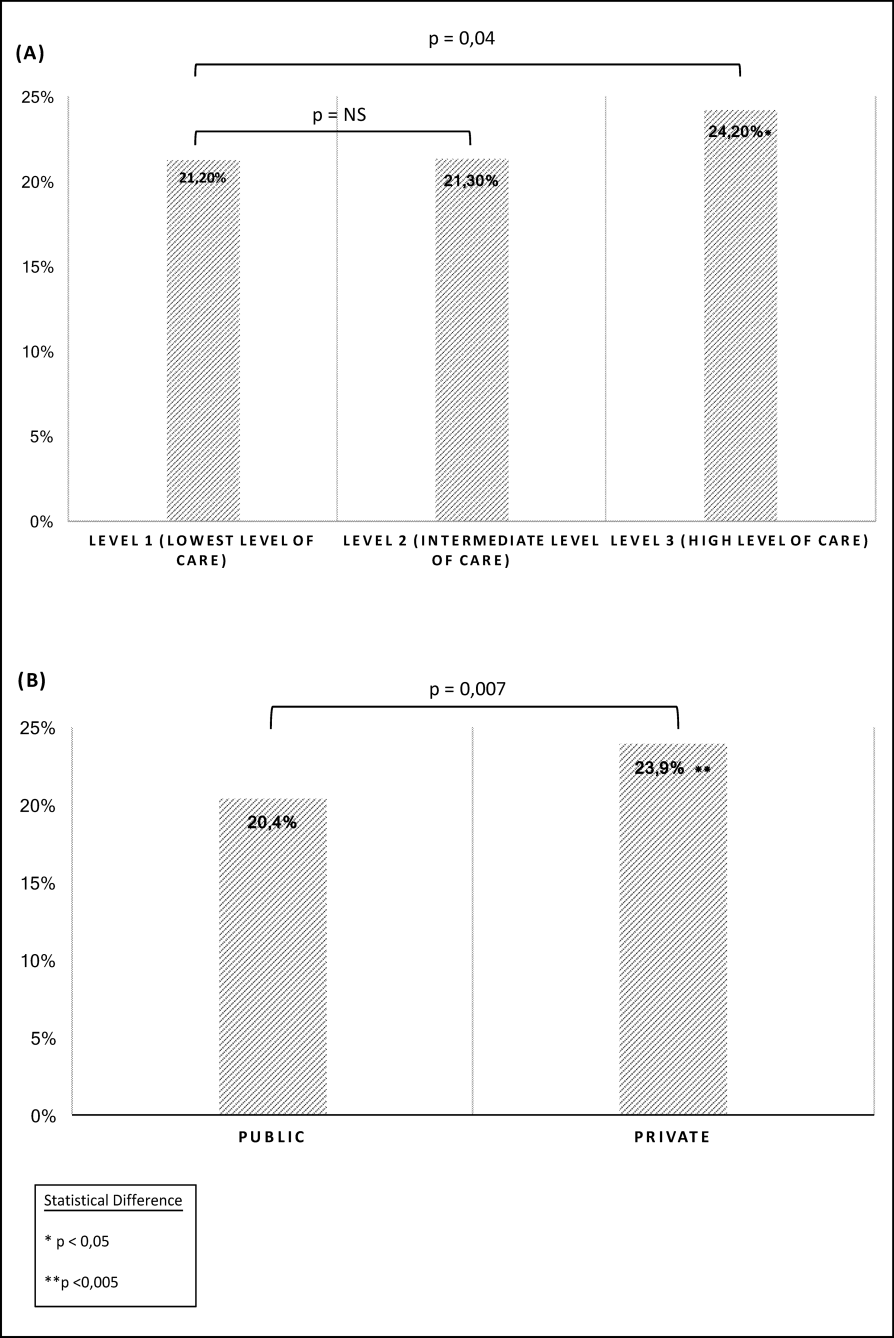
Caesarean delivery rate according to the level of care (A) and status (B) of the maternity facilities between 2012 and 2015 in the Provence-Alpes-Côte d’Azur.

There was a significant difference in caesarean delivery rates according to the status of the maternity facility. The rate was higher in private facilities compared to public maternity facilities. Similarly, the caesarean rate was significantly higher in type III maternity facilities compared to type I maternity facilities. “Fig 1”.

### Univariate analysis of factors associated with the overall caesarean rate

The following factors concerning characteristics of maternity facility and professional practices were associated with the caesarean rate. Data are reported in “Table 1”.

**Table 1:** Univariate analysis of factors associated with the caesarean rate in the Provence-Alpes-Côte d’Azur Region between 2012 and 2015 (CS = Caesarean Section).

Studied factors between 2012 and 2015		n (number of maternities)	CS (%) Mean caesarean rate in maternity facilities	p
**Size of town**				
Small town (< 20 000 hts)	Yes	6	1	**0,03**
	No	34	21,5 +/- 3,6	
Medium town (20–200 000 hts)	Yes	24	20,4 +/- 3,2	
	No	16	22,9 +/- 3,2	
Large town (>200 000 hts)	Yes	30	24,2 +/- 3,2	
	No	10	20,5 +/- 3,7	
**Protocols**				
Fetal heart rhythm abnormalities	Yes	23	21,2 +/- 3,9	0,65
	No	17	1	
Gestational diabetes and fetal macrosomia	Yes	27	21,3 +/- 3,5	0,76
	No	13	1	
Occiput posterior fetal position	Yes	4	21,6 +/- 3,9	0,26
	No	36	1	
Multiple pregnancies	Yes	11	21,3 +/- 2,4	0,90
	No	29	1	
Breach presentation	Yes	15	21,6 +/- 2,5	0,84
	No	25	1	
Uterine Scarring	Yes	23	21,3 +/- 4,4	0,92
	No	17	1	
Induction methods	Yes	28	20,7 +/- 3,4	0,07
	No	12	1	
Elective cesarean section	Yes	4	23,3 +/- 4,7	0,34
	No	36	1	
**Daily obstetrical staff meetings**				
	Yes	17	20,6 +/- 3,4	0,27
	No	23	1	
**Assessment of professional practices**				
Morbidity-mortality reviews	Yes	35	21,8 +/- 7,1	0,41
	No	5	1	
Patient feedback	Yes	23	21,8 +/- 4,4	0,46
	No	17	1	
Clinical pathway	Yes	9	21,6 +/- 4,3	0,85
	No	31	1	
Relevance reviews	Yes	15	21,9 +/- 4,3	0,49
	No	25	1	
Audit related to caesarean rates	Yes	21	22,8 +/- 3,5	**0,01**
	No	19	1	
**Second line monitoring**				
Scalp PH	Yes	14	22,1 +/- 4,3	0,43
	No	26	1	
Lactate PH	Yes	17	20,1 +/- 3,5	0,07
	No	23	1	
**Assistance provided during caesarean deliveries**				
Scrub nurses	Yes	24	21,5 +/- 3,5	0,83
	No	16	21,3 +/- 4,2	
Midwives	Yes	9	22,1 +/- 3,8	0,55
	No	31	21,2 +/- 4,3	
Foundation doctors	Yes	15	20,2 +/- 4,1	0,13
	No	25	22,1 +/- 3,3	
None			1	
**Maternity offering training to trainees doctors**				
	Yes	23	20,3 +/- 2,8	**0,03**
	No	17	1	

#### Size of the town

A significantly higher caesarean rate was observed in large towns compared to small towns (p<0.01 in 2012 and 2013, p = 0.05 in 2014 and p = 0.02 in 2015).

#### Assessment of professional practices

Maternity facilities that assessed an audit on the caesarean delivery rate had a significantly lower caesarean rate compared to those who did not (p<0.01 in 2012 and 2013, p = 0.05 in 2014 and p = 0.02 in 2015). The association was not observed with assessments of practices on other topics (Morbidity-mortality reviews, patient feedback, clinical pathways, relevance reviews and non-assessment of professional practices).

#### Training to trainee doctors

The maternity facilities offering training to trainee doctors had a significantly lower caesarean delivery rate compared to those who did not (p<0.03 in 2012, p = 0.05 in 2013, p = 0.04 in 2014 and p = 0.02 in 2015).

#### Induction protocol

In 2012, the use of an induction protocol was also associated with a lower caesarean delivery rate compared to those who did not use (p = 0.05), although this relationship was not observed between 2013 and 2015.

#### Use of scalp lactate

The same situation was observed for the use of scalp lactate in 2012 and 2013 compared to those who did not use (p<0.05). However, this significant relationship was not observed in the following years, or with the use of scalp pH.

#### Delivery room practitioners

There were higher caesarean delivery rates for practitioners working in the delivery room (p = 0.01 in 2012, 2013 and 2015 and p = 0.04 in 2014) and for practitioners on call at night and weekends (p = 0.01 in 2012, 2013 and 2015 and p = 0.03 in 2014) compared to those who did not have these activities. Data are reported in “Table 2”.

**Table 2:** Univariate analysis of correlations between practitioner characteristics and caesarean delivery rates.

	2012		2013		2014		2015	
	R	p	R	p	R	p	R	p
**Participation of each practitioner**								
Delivery room	0,49	0,01	0,41	0,01	0,32	0,04	0,45	0,01
On call at night and weekends	0,49	0,01	0,41	0,01	0,35	0,03	0,46	0,01
**Age of each practitioner**								
30–40 age	0,23	0,16	0,20	0,22	-0,02	0,89	0,14	0,38
40–50 age	0,28	0,08	0,25	0,12	0,21	0,18	0,27	0,09
50–60 age	0,41	0,01	0,33	0,04	0,28	0,05	0,38	0,02
60–70 age	0,36	0,02	0,30	0,05	0,39	0,01	0,38	0,02
**Gender of each practitioner**								
Female practitioner	0,18	0,27	0,23	0,15	0,05	0,75	0,17	0,3
Male practitioner	0,52	0,01	0,37	0,02	0,40	0,01	0,48	0,001

#### Practitioner characteristics

There was a significant relationship between caesarean delivery rate and the age and gender of the practitioners. The caesarean delivery rate was significantly higher for male practitioners (p<0.001 for each year from 2012 to 2015) and for practitioners over the age of 50 (p<0.05 from 2012 to 2015 for practitioners aged 50–60 and 60–70 compared with the 30–40 age bracket). Data are reported in “Table 2”.

There was no statistical link between the caesarean delivery rate and the use of other protocols for situations where there is a risk of a caesarean delivery.

Similarly, there was no significant association between the caesarean delivery rate and the holding of daily obstetrical staff meetings, the distance between the maternity facility and a university hospital, the availability of surgical assistance and the type of assistance.

### Multivariate analysis

The variables analysed were: status of the maternity facility, level of care of the maternity facility, audit focusing on caesarean deliveries, scalp lactate, surgical assistance, practitioners working in the delivery room and on call, the size of the town, the use of an induction protocol, training to trainee doctors and the age and gender of practitioners.

After a stepwise regression, the variables associated with a lower rate in the caesarean delivery rate were assessments of professional practices concerning caesarean delivery (19.83%, β = - 2.48, p = 0.03 over the four years) and the provision of training to trainee doctors at the maternity facility (20.28%, β = - 1.08, p = 0.04 over the four years). Larger towns were significantly associated with a higher caesarean delivery rate (β = 3.09, p = 0.02). Data are reported in “Table 3”.

**Table 3:** Multivariate analysis.

Studied factors between 2012 and 2015		ß	p
**Status of maternity**			
Public		1	0,72
Private–for profite		1,24	
Private–not for profit		1,02	
**Level of care of maternity**			
Type I (lowest level of care)		1	0,79
Type II (Intermediate level of care)		0,62	
Type III (highest level of care)		1,11	
**Size of town**			
Small town (<20 000)		1	**0,02**
Medium town (20–200 000)		1,2	
Large town (>200 000)		3,09	
**Protocol**			
Induction methods	Yes	-0,79	0,05
	No	1	
**Maternity**			
Offering training to trainees doctors	Yes	-1,08	**0,04**
	No	1	
**Assessment of professional practices (audit)**			
Focusing on the caesarean delivery rate	Yes	-2,48	**0,03**
	No	1	
**Second line monitoring**			
Lactate PH	Yes	-1,86	0,09
	No	1	
**Participation of each practitioner**			
Delivery room		0,39	0,18
On call at night and weekends		0,41	
None		1	
**Age of each practitioner**			
Age 30–40		1	0,97
Age 40–50		0,28	
Age 50–60		0,43	
Age 60–70		0,43	
**Gender of each practitioner**			
Female practitioner		1	0,55
Male practitioner		0,27	

## Discussion

After analysing the 55 097 caesarean sections realised between 2012 and 2015 in south of France, performing audits in relation to caesarean deliveries were associated with lower caesarean delivery rates. Moreover, to our knowledge, no study has yet shown that maternity facilities offering training to trainee doctors had a significantly lower caesarean delivery rate.

In our study, we observed a significantly higher caesarean delivery rate in private and in type III (maximum care level) maternity facilities. The higher caesarean delivery rates in level 3 maternity facilities can be explained by the overmedicalisation of pregnancies, less regard for physiology and the higher number of problem pregnancies managed there. As for the association between a higher caesarean delivery rate and private status, several hypotheses can be put forward: the tendency for professionals to avoid a vaginal delivery for pregnancies with a greater likelihood of neonatal and obstetric complications, and the higher frequency of scheduled caesareans (uterine scarring) and elective caesareans in this type of facility, are no doubt related to the organisational constraints of this type of facility [13,14].

Our results showed that audits concerning caesarean deliveries could reduce the caesarean delivery rate. It has been established that when precise information is given on the topic and feedback is provided to the professionals there is an impact on daily practices [15]. In our study, some of these audits were focused on groups contributing the most to the overall caesarean section rate of the Robson classification (groups 1, 2 and 5) [16–18]. Unfortunately, the Robson classification could not be evaluated in our study owing to some currently unavailable regional data and to evaluate the factors that could influence groups of this classification.

Similarly, a lower rate of caesarean section was observed in maternity wards participating in the training to doctors. Several interpretations can be made concerning this studied variable: The lower rate of caesarean section is more likely to be related to the university hospital environment and not just to the training of trainee doctors. Indeed, we have observed that a university hospital environment allows multiple consultations (obstetrical staff meetings, multidisciplinary consultations, assessment of professional practices). This type of structure delivers and organizes a university education, and encourages maternity facilities to take care to update the literature, apply clinical practice recommendations and analyse situations where management is difficult. Nevertheless, in our study, 17 maternity facilities (40%) offering training to trainee doctors. Of these maternity facilities, only two were university hospitals. The other 15 did not systematically perform obstetrical staff meetings, multidisciplinary consultations and assessment of professional practices. So, teaching to trainees doctors could be probably more an indicator of quality of caesarean practices. Maternities with trainees doctors organize teaching and therefore, need to actualize their knowledge. Feedback with trainees doctors and the team could also participate to caesarean section decrease.

Concerning the use of induction protocols, induction indications and methods can be governed by protocols to address the potential risks involved, which include the possible increase in the risk of caesarean delivery, even if they do not affect the caesarean delivery rate in our study (p = 0.05) [19]. The use of scalp lactate, associated with a decrease in the caesarean delivery rate, observed in 2012 and 2013 in univariate analysis but not in multivariate analysis, is difficult to interpret that it was a binary variable so we did not have access to the frequency of scalp lactate use in each maternity unit. There was also a relationship between the size of the town and the caesarean delivery rate: the higher caesarean rate in large towns is most likely due to the greater diversity of maternity facility care levels and the greater number of type 3 facilities.

In our study, performing audits on caesarean delivery is associated with a 2% decrease in the CS rate. According to the last perinatal survey in 2016 [2], the 2% represents a decrease of 32 000 caesareans section in France and 556 000 caesareans around the world, according tho WHO figures [4].

According to Villar et al [20], caesarean delivery is associated with a higher risk of severe maternal and neonatal morbidity and mortality. A decrease of 2% could allow a significant decrease in these potential complications and the economic cost of healthcare.

There is an information bias in our study as the data are from 2012 to 2015, but the questionnaire data reflect 2017 practices. Nevertheless, the 2016 and 2017 data were not yet available when the study was conducted and no questionnaire evaluating practices that could influence the caesarean delivery rate had been completed before 2017. Finally, the accuracy of our data, derived from the French National Computerized Medical Information System (PMSI), can be criticized.

Identifying factors affecting the caesarean rate can be useful for implementing specific measures to reduce the caesarean delivery rate [21]. A number of initiatives have been implemented by various obstetrics teams across the world: introducing an obstetrics audit has already proved to be effective in reducing the rate of caesarean deliveries, particularly in France (daily obstetrical staff meetings to validate the indications for caesarean deliveries) [15], and in Quebec (assessment of professional practices including a review of the indications for each practitioner’s caesarean) [22]. The impact of implementing a 9-item checklist to manage labour at maternity facilities has proved to be effective in Sweden, as there was a significant decrease in the caesarean delivery rate (from 20% to 11%) between 2006 and 2015 [23]. In France in 2016, Lembrouck et al. suggested daily discussions on obstetrics cases, wider acceptance of vaginal deliveries and the continuous assessment of practices after observing a decrease in the caesarean delivery rate from 23.3% to 19.7%, p = 0.05, in their maternity facility [24]. All these authors agree that relevant and accurate tools, such as clinical audits, continuous assessments of professional practices regarding individual caesarean rates and using checklists prior to performing a caesarean are beneficial in reducing the caesarean delivery rate [25].

It would be interesting to prospectively evaluate the use of such approaches, especially in groups where caesarean rates are the highest of the Robson Classification, because of their objective and standardized characteristics.

## Conclusion

In our study, the caesarean delivery rate was higher in private facilities and in level 3 maternity facilities. Perform audits on caesarean delivery could reduce the caesarean delivery rate. Teaching to trainees doctors could be an indicator of quality of caesarean practices. Indeed, doctors organize teaching and therefore, need to actualize their knowledge and practice recommendations. It would be a good thing that a maximum of maternities has an agreement to receive trainees doctors in a process of reducing caesarean section rate. Authors concur that relevant and accurate tools are now needed to reduce the caesarean delivery rate. An interventional approach in an extensive prospective trial would be very appropriate, recalling and encouraging compliance with good practices.
